# General Overview of Nontuberculous Mycobacteria Opportunistic Pathogens: *Mycobacterium avium* and *Mycobacterium abscessus*

**DOI:** 10.3390/jcm9082541

**Published:** 2020-08-06

**Authors:** Kimberly To, Ruoqiong Cao, Aram Yegiazaryan, James Owens, Vishwanath Venketaraman

**Affiliations:** 1 Graduate College of Biomedical Sciences, Western University of Health Sciences, Pomona, CA 91766-1854, USA; kimberly.to@westernu.edu (K.T.); aram.yegiazaryan@westernu.edu (A.Y.); 2 Department of Basic Medical Sciences, College of Osteopathic Medicine of the Pacific, Western University of Health Sciences, Pomona, CA 91766-1854, USA; rcao@westernu.edu (R.C.); jowens@westernu.edu (J.O.)

**Keywords:** Nontuberculous mycobacteria, *Mycobacterium avium*, *Mycobacterium abscessus*, Mycobacterium avium complex, *M. tuberculosis*, bronchiectasis, lymphadenitis, immunocompromised, Azithromycin, macrolides

## Abstract

Nontuberculous mycobacteria (NTM) are emerging human pathogens, causing a wide range of clinical diseases affecting individuals who are immunocompromised and who have underlying health conditions. NTM are ubiquitous in the environment, with certain species causing opportunistic infection in humans, including *Mycobacterium avium* and *Mycobacterium abscessus*. The incidence and prevalence of NTM infections are rising globally, especially in developed countries with declining incidence rates of *M. tuberculosis* infection. *Mycobacterium avium*, a slow-growing mycobacterium, is associated with *Mycobacterium avium* complex (MAC) infections that can cause chronic pulmonary disease, disseminated disease, as well as lymphadenitis. *M. abscessus* infections are considered one of the most antibiotic-resistant mycobacteria and are associated with pulmonary disease, especially cystic fibrosis, as well as contaminated traumatic skin wounds, postsurgical soft tissue infections, and healthcare-associated infections (HAI). Clinical manifestations of diseases depend on the interaction of the host’s immune response and the specific mycobacterial species. This review will give a general overview of the general characteristics, vulnerable populations most at risk, pathogenesis, treatment, and prevention for infections caused by *Mycobacterium avium*, in the context of MAC, and *M. abscessus.*

## 1. Introduction

Nontuberculous mycobacteria (NTM) are a group of bacteria under the genus *Mycobacterium,* which includes *Mycobacterium tuberculosis,* comprising more than 172 species with many unique virulence characteristics (see Table 1 for examples of pathogenic species). NTM are found ubiquitously in the environment and present in water sources, soil, domestic and wild animals, and milk and food produce. NTM are opportunistic pathogens to animals and humans, including fish and poultry [1]. As opposed to tuberculosis caused by *M. tuberculosis* infection, NTM infection reporting is not mandatory; therefore, the incidence and prevalence of the different species of NTM are difficult to determine. However, the prevalence of NTM infection is growing in the United States, Europe, and other developed countries in the Western world. The increased occurrences of NTM infections are associated with declining tuberculosis rates in areas of higher socioeconomic standards. In a systematic review of 22 studies between 1946 and 2014, most studies overall found an 81% declining tuberculosis (TB) incidence rate, while NTM disease rose by 94% in almost every geographic area [2]. Their importance is due to their growing emergence as human pathogens causing opportunistic infections in severely immune-compromised individuals, people with congenital or acquired anatomical lung diseases, and healthcare-associated infections [1]. However, there is also a noted increased incidence in NTM disease in people who are not immunocompromised and without any preexisting lung diseases. One study found that 18% of MAC pulmonary infections were from people without predisposing conditions, similar to the other literature on this group [3]. Another study found that, for this group, they accounted for 20% of all clinical NTM infections; they also tended to have a delayed diagnosis and a high recurrence rate [4].

NTM infection mainly presents four distinct clinical diseases in humans: (1) chronic pulmonary disease; (2) disseminated disease in severely immunocompromised patients; (3) skin and soft tissue infection; and (4) superficial lymphadenitis, especially cervical lymphadenitis in children. Pulmonary disease accounts for 80–90% of NTM-associated diseases [5]. The most common NTM species causing human disease are the slow-growing mycobacteria *Mycobacterium avium* in the *Mycobacterium avium* complex (MAC), the rapid-growing *M. abscessus,* and *M. kansasii*. In this review article, we will discuss the vulnerable populations affected by these opportunistic infections, the pathogenesis, treatment, and prevention of infections caused by *Mycobacterium avium*, with a focus on the *Mycobacterium avium* complex (MAC), as well as infections caused by *Mycobacterium abscessus.*

## 2. Mycobacterium avium

### 2.1. General Characteristics

*Mycobacterium avium* complex (MAC) infections are caused by a group of mycobacteria consisting of *M. avium*, *M. intracellulare*, and the newly described mycobacterium species, *Mycobacterium chimaera*, which are all acid-fast slow-growing mycobacteria, classified as non-chromogens in group III of the Runyon classification of NTM. MAC initially included only *M. avium* and *M. intracellulare*, but with new genetic sequencing technology, phenotypic and genotypic tests have identified additional species. To date, MAC contains nine species of slow-growing mycobacteria: *M. avium, M. intracellulare, M. chimaera, M. colombiense, M. marseillense, M. timonense, M. boucherdurhonense, M. vulneris, M. arosiense*, and a small group of unclassified, “MAC others” [6]. Recently, *M. chimaera* was implicated in worldwide outbreaks in five countries including the USA, Europe, and Australia, due to contaminated heater–cooler unit water tanks in patients that underwent open-heart surgery, such as coronary artery bypass grafting [7]. Investigations found the culprit was contamination from the Stöckert 3T heating and cooling unit manufactured by LivaNova PLC in London [8]. The sequence of the 16-23S internal spacer (ITS) region of *M. chimera* corresponds to the sequevar MAC-A compared to the *M. intracellulare* sequevar MIN-A (DSMZ 43223) by 20 nucleotide (nt) mismatches. Additionally, the 16S rRNA gene sequence between the two species only has a 1 nt mismatch. Since the sequence of 16S rDNA is considered the approved standard for identifying NTM species, *M. chimaera* is usually misreported as *M. intracellulare* [9,10]. Traditionally, standard molecular genetic tools in clinical microbiology laboratories did not differentiate MAC members and only gave a rough classification of *M. avium*, *M. intracelllulare*, or MAC as a whole since treatment is the same for all MAC species. Detailed genotyping of MAC species was limited to specialized research laboratories that can perform PCR of several genes such as rpo or hsp65 and ITS [11]. However, due to these recent outbreaks associated with cardiac surgery, clinical laboratories are now mandated to differentiate *M. chimaera* from other MAC species [7].

The most clinically significant organism for human disease within MAC is *Mycobacterium avium*, with four distinct subspecies. This group of bacteria ranges from environmental bacteria that can cause opportunistic infections in immunosuppressed individuals to pathogens for birds and other animals. The characteristics of the four distinct species of *M. avium* are discussed. *M. avium* subsp. *Hominissus* (MAH) is an opportunistic environmental pathogen for humans, swine, and other animals that are found in soil and water. Drinking water and tap aerosols are considered the primary sources for infection in humans. MAH is clinically the most relevant organism within MAC for humans and a major pathogen for individuals with deficient T cell immunity. Before the development of antiviral therapy, in the early years of the Acquired Immunodeficiency Syndrome (AIDS) epidemic, 20–50% of severely immunocompromised AIDS patients had disseminated infections with MAC organisms, now identified as MAH infections [12]. MAH is still a threat to newly diagnosed HIV patients and individuals with no access to antiviral therapy or for those that antiviral therapy is ineffective [13]. In immunocompromised individuals, MAH can cause pulmonary infections, cervical infections, mainly in children, and soft tissue infections. *M. avium* subsp. *Paratuberculosis* (MAP) is a widely known pathogen that causes paratuberculosis, also known as John’s disease, primarily affecting ruminants, causing chronic progressive infection in the small intestine. Non-ruminants, and non-human primates can be infected with MAP as well. MAP has a worldwide distribution with transmission mainly by the fecal–oral route via pasture, milk, and water contaminated with feces [14,15]. In humans, MAP has been proposed to be linked to Crohn’s disease, due to a meta-analysis demonstrating their association, however, the association remains controversial and inconclusive [16]. *M. avium* subsp. *Avium* (MAA) mainly causes a tuberculosis-like disease in birds, the main reservoir of MAA. Avian TB is a chronic wasting disease, showing little signs of infection. A variety of mammals, but mostly pigs and cattle, can become infected with MAA [17]. Transmission is mainly from birds to susceptible animals via ingestion or through the environment with fecal contamination [18]. *M. avium* subsp. *Silvaticum* (MAS) is taxonomically similar to MAA and is a pathogen to the wood pigeon, causing TB-like disease. Some experimental studies also show that, in mammalian species, MAS can cause chronic enteritis [19].

### 2.2. Vulnerable Populations to Mycobacterium avium Complex (MAC) Infections

The pathogenesis of *M. avium* depends on its ability to colonize intestinal or respiratory mucosa, evade protective barriers, and resist intracellular killing in macrophages. The disease normally does not develop in most people; however, immunocompromised patients or patients with pre-existing conditions are at higher risk of MAC infections. There are three types of MAC infections that can occur: (1) pulmonary MAC infections, (2) disseminated MAC infections, and (3) MAC-associated lymphadenitis [20].

Pulmonary MAC infections affecting lungs are the most common type. These infections mainly affect patients with chronic lung diseases, such as chronic obstructive pulmonary disease (COPD), chronic bronchitis, bronchiectasis, cystic fibrosis, and lung cancer. Pulmonary infections are likely acquired through inhalation of aerosols from the natural or institutional water systems. The fibrocavitary disease form of MAC is common in older male smokers with chronic pulmonary symptoms. The nodular/bronchiectatic disease form is most common in nonsmoking women over the age of 50 with chronic lung disease [20]. Another predisposition for the nodular/bronchiectatic disease form is particular body morphotypes with similar clinical characteristics including scoliosis, pectus excavatum, mitral valve prolapse, and joint hypermobility [21]. These pulmonary infections are characterized as a localized infection; however, it can spread to submucosal tissue, enter the bloodstream, and infect other organs and tissues. This results in disseminated MAC infections and usually affects patients with HIV/AIDS.

Disseminated MAC (DMAC) infections usually occur in patients in advanced stages of HIV/AIDS when blood CD4+ T cell counts are lower than 50/mm^3^, unlike *M. tuberculosis* that can infect patients at any stage of AIDS [22]. MAC is most likely acquired by the gastrointestinal tract in HIV-infected patients [23]. In the developed world, DMAC is the most frequent opportunistic bacterial infection in HIV patients with an annual occurrence of 10–20% in those who have AIDS [24]. Before the availability of antiretroviral therapy (ART), *M. avium* was the etiologic agent in >95% of individuals living with HIV with advanced immunosuppression. In recent years, with the availability of antiretroviral therapy (ART), the rate of DMAC has fallen substantially [13]. However, adults with HIV and advanced immunosuppression who are not receiving or unable to tolerate ART are still at risk of disseminated MAC. Without antiretroviral therapy or prophylactic antibiotics, the incidence of DMAC among HIV-infected patients with CD4 counts less than 100/mm^3^ is approximately 20% [24].

MAC can also cause lymphadenitis, mainly cervical lymphadenitis in immunocompromised children under five years old. However, one study indicated that it primarily affects healthy children under 15 years old with normal immune systems, and is an underrecognized cause of lymphadenitis in young children [25,26]. There are also rare Mendelian genetic susceptibilities to mycobacterial disease related to IFN-γ production deficiencies or receptor abnormalities [27,28]. IFN-γ or IL-12 receptor deficiencies cause approximately 80% of the genetically diagnosed cases. These rare monogenic disorders resulting in susceptibility to NTM infections are grouped together as Mendelian Susceptibility to Mycobacteria Disease (MSMD) conditions. Most Mendelian susceptibilities for NTM infections are mutations in genes encoding cytokines, receptors and downstream signaling-transducing proteins of the 1L-12/IFN-γ axis. Mutations in STAT1, a transcription factor also results in an impaired response to type I (IFN-α/β), type II (IFN-γ) or type III (IFNλ-1/2/3) interferons [29]. Mutations in the interferon regulatory factor (IRF)-8 also undermine production of IL-12 in response to IFN-γ [30]. Mutations in interferon-stimulated gene (ISG)-15, whose product is involved in IFN-γ production by T and natural killer (NK) cells, likewise increase susceptibility to NTM infections [31]. The autosomal dominant deficiency of the transcription factor GATA-2 also carries a significant risk to NTM infections; this contributes to a deficiency of mononuclear phagocytes [32,33]. Auto-antibodies to IFN-γ, use of TNF-α inhibitors, mutations in RAR-related Orphan Receptor (RORC), and CD4+ T cell deficiency are also factors contributing to host susceptibility [20,34]. Phagocyte defects, such as chronic granulomatous disease (CGD), which impair the respiratory burst due to the lack of a functional NADPH oxidase, have also been shown to produce systemic complications in patients receiving the bacillus Calmette–Guerin (BCG) vaccine [35].

Immunosuppressive therapies taken by transplant recipients or patients with immune-mediated inflammatory diseases also lead to a higher risk of NTM infections. The use of corticosteroids, TNF-α treatment, or TNF-α inhibitors significantly increase the risk of developing pulmonary or disseminated NTM infections. One study found that immune-mediated inflammatory disease patients receiving anti-TNF-α treatment had elevated rates of mycobacterial disease and that NTM disease was associated with rheumatoid arthritis (RA) [36]. For organ transplant recipients taking a variety of immunosuppressive mediations such as calcineurin inhibitors (tacrolimus and cyclosporine), mammalian target of rapamycin (mTOR) inhibitors (sirolimus), and prednisone, in one case study, these were shown to be one of the most important risk factors of NTM infection, accounting for 55.9% of cases (19/34) [37].

### 2.3. Pathogenesis of Mycobacterium Avium/MAC

#### 2.3.1. Invasion and Adherence of Mucosal surfaces

These mycobacteria are acquired through the respiratory or the intestinal route, able to invade mucosal epithelial cells and translocate across the intestinal and respiratory mucosa to then infect phagocytic cells [38]. Studies have shown that *M*. *avium* can resist the acidic pH conditions of the stomach and were able to gain access to the intestinal lumen. *M. avium* administered orally are isolated from the jejunum, ascending colon, and the majority in the terminal ileum [39]. Other studies also show *M. avium* preferentially entering the terminal ileum via enterocytes of both Peyer’s patches and outside the Peyer’s region [38].

Another mechanism associated with *M. avium*’s ability to evade epithelial cells is their interaction with fibronectin-attachment protein (FAP), allowing them to utilize fibronectin like a “bridge” to attach to integrin receptors found on the cell membrane of mucosal cells [40]. Middleton et al. found that MAC can adhere to the extracellular matrix (ECM) in areas of epithelial damage via fibronectin attachment protein (FAP) and to the mucus via another adhesin [41]. Hemagglutinin binding protein has also been linked to the *M. avium* invasion of epithelial cells, although direct evidence is lacking [42].

Once attached to the mucosal cells, *M. avium* triggers uptake on the apical surface, associated with a cytoskeleton reorganization and the phosphorylation of proteins [43]. The precise receptors utilized by the bacterium to infiltrate the mucosal cell are unknown. The entry of *M. avium* is not associated with an inflammatory response [44]. However, there is an association with the suppression of chemokines, interleukin-8 (IL-8), and RANTES in the cells of the epithelium, all of which are mechanisms to reduce the host response to the infection [45]. Mechanisms for the bacterium to exit the polarized intestinal cells are not known at this time, but it has been recognized that the bacterium leaves the mucosal epithelial cell with a phenotype that is significantly more effective with for the invasion of macrophages [46]. *M. avium* may also bypass the mucosa, and then locate to lymph nodes of the mesentery, where it survives until the immune system is compromised, such as with AIDS patients [40].

The pathway for infection of the respiratory tract is less well known than the intestinal route, but initiation is most likely through inhalation of aerosols of the bacteria. *M. avium* respiratory infections usually only affect patients with chronic lung disease, and their establishment of the infection could be due to the development of biofilms, which *M. avium* is known to establish in municipal water systems [47]. Once *M. avium* contacts the alveolar space, it was found they are capable of invading type II alveolar epithelial cells and replicating within these cells [48].

#### 2.3.2. Interaction with Phagocytes

The primary host cells for *M. avium* and most mycobacteria are in mononuclear phagocytes such as monocytes and macrophages. The bacterium can gain access into monocytes and macrophages by several different receptors: complement receptors, mannose receptors, type A scavenger receptors, and in hosts with mycobacteria-specific antibodies, Fc-gamma receptors [49]. The use of these receptor types is most likely redundant since mice lacking complement receptor CR3 and CR4 were still able to establish an infection indistinguishable from immunocompetent control mice [50]. Once bound to the cell, the mycobacterium is taken up in primary phagosomes that fuse with vacuoles inside the macrophages’ cytoplasm. An infection occurs when it survives and proliferates within vacuoles as an intracellular pathogen. Its ability to survive is through immune evasion mechanisms such as the inhibition of phagosome–lysosome fusion, the inhibition of the acidification of the phagocytic vacuole, the disruption of the actin microfilament cytoskeleton, the induction of NTM-related genes to enhance replication, and a shift in metabolism to a more anaerobic intracellular environment [51,52,53,54,55].

#### 2.3.3. Host Defense and Immune Response

The host defense against *M. avium* involves both the nonspecific (innate) and specific (adaptive) immune system working together and secreting cytokines in response to *M. avium*. The innate immune response contains natural killer (NK) cells, which are critical components of this response, and have been confirmed to play a part in the nonspecific immune response against *M. avium* in mice and human systems [56,57]. Mice with dysfunctional natural killer cells were found to be more prone to an *M. avium* infection [58]. The host’s specific immune response to *M. avium* has been shown to be CD4+ T lymphocyte-dependent and based on the generation of IFN-γ. CD8+ T cells roles are not well defined, but studies have shown that CD4+, but not CD8 T cells are required for adaptive immunity against *M. avium* [59,60].

Macrophages are the central players in the defense against *M. avium*. Macrophages stimulated with cytokines TNF-α, IFN-γ, and GM-CSF can control infection [61] and were confirmed in experimental mice [59]. IL-12 is also a key cytokine in the host defense against mycobacteria. Infected macrophages respond with the production of IL-12, activating NK cells, and T lymphocytes to proliferate and secrete cytokines [62]. NK cells secrete TNF-α, IFN-γ, and GM-CSF in response to *M. avium* [63]. When stimulated by NK cell products, infected macrophages were able to control the intracellular infection [64]. Although IL-12 is initially secreted from infected macrophages, there is progressive suppression of IL-12 and an inverse relationship with the period of infection [62]. The exact mechanisms of macrophages killing *M. avium* are less known, but preliminary research suggests that the activation of macrophages gives rise to changes in the vacuoles of the intracellular environment [65].

### 2.4. Treatment of Mycobacterium avium/MAC Infections

The *Mycobacterium avium* complex (MAC) consists of most of the common pulmonary NTM pathogens that are heavily linked to the causes of human disease in almost all regions of the world [66]. As previously mentioned, MAC infections consist of (1) pulmonary MAC infections, (2) disseminated MAC infections, and (3) MAC-associated lymphadenitis. According to the American Thoracic Society, Pulmonary NTM infections are categorized even further based on radiologic criteria [5].

#### 2.4.1. Pulmonary MAC Infections

The three categories of pulmonary MAC infections are as follows: (1) noncavitary nodular bronchiectatic disease, patients who have bronchiectasis clusters with less than 5-mm nodules; (2) cavitary nodular bronchiectatic disease, patients who also present with lung cavities in addition to bronchiectasis; and (3) fibrocavitary disease, patients with cavities, fibrosis, and/or pleural involvement over adjacent areas.

The treatment of pulmonary MAC infections consists of antimicrobial therapy, particularly macrolides, followed by close follow-up monitoring for up to a year. For patients with noncavitary or mild/moderate bronchiectatic disease, the current guidelines recommend a three-drug, macrolide-based regimen (including a macrolide and ethambutol) for macrolide-susceptible MAC pulmonary disease.

For example, a three-drug regimen of azithromycin (500 mg 3×/week), rifampin (600 mg 3×/week), and ethambutol (25 mg/kg 3×/week) can be used (see Table 2). An azithromycin-based regimen is recommended over clarithromycin-based regimens due to its better tolerance, less drug interactions, lower pill burden, and equal efficacy [67].

In more severe cases, such as in cavitary or advanced/severe nodular bronchiectatic disease, instead of 3×/week, a daily regimen is recommended. For example, azithromycin (250 to 500 mg daily), rifampin (600 mg daily), and ethambutol (15 mg/kg daily) can be used along with parenteral amikacin or streptomycin in the initial treatment [68]. It is recommended that parenteral amikacin (10 to 15 mg/kg 3×/week) or streptomycin be used in the initial first 2–3 months of therapy for patients with cavitary, severe/advanced bronchiectatic MAC pulmonary disease, or macrolide-resistant MAC [67]. Patients with macrolide-susceptible MAC pulmonary disease should receive treatment for at least 12 months after culture conversion.

For patients with no positive outcome after 6 months with the guideline-based therapy, an amikacin liposome inhalation suspension (ALIS) treatment should be utilized in addition to their current treatment regimen. ALIS has demonstrated efficacy and safety when added to guideline-based therapy and is currently approved by the United States Food and Drug Administration (FDA) for treatment for refractory MAC pulmonary disease [69,70]. Surgery should also be considered from a risk/benefit perspective for certain patients who have not responded within 6 months [71]. The American Thoracic Society (ATS) and the Infectious Diseases Society of America (IDSA) recommend that surgical resection of MAC lung disease should be limited to patients with an adequate cardiopulmonary reserve to withstand partial or complete lung resection along with a multidrug treatment regimen. Additionally, surgery for MAC lung disease should be performed in centers with expertise on both the medical and surgical management of NTM diseases. Surgical resection of solitary pulmonary nodules from MAC is considered curative [5]. One study on treatment outcomes for 70 patients who had adjunctive pulmonary resections of NTM disease, had successful treatment outcomes for over 80% of its patients overall, although the post-operative outcomes and complications were relatively high. It is also important to note that the outcomes and the complications vary by the severity of the disease and type of surgery performed [71].

It is also important to note that the Clinical and Laboratory Standards Institute (CLSI) guidelines recommend antimycobacterial susceptibility testing (AST) for all slow-growing NTM infections, including MAC on all clinically significant isolates for proper antibiotic selection for a treatment regimen [72]. AST is performed for drugs which have a clear correlation between in vitro and in vivo outcomes. MAC has a clear correlation between the baseline macrolide susceptibility of the causative strain and the treatment outcomes with macrolide–ethambutol–rifampicin regimens [73,74]. The acquired macrolide resistance for MAC is from point mutations in the 23S rRNA(*rrl*) gene [75,76], while the acquired amikacin resistance is due to mutations in the 16S rRNA (*rrs*) gene [77]. The breakpoints for resistance are a MIC ≥ 64 μg/mL for parenteral amikacin and ≥128 μg/mL for amikacin liposome inhalation suspension (ALIS) [78]. Finding MICs above these breakpoints would require a cessation of intravenous or nebulized amikacin therapy [69]. The CLSI have also provided tentative breakpoints for linezolid and moxifloxacin, although in vitro–in vivo correlations have not been established [78].

#### 2.4.2. Disseminated MAC Infection

Disseminated MAC infections spread to the rest of the body instead of staying localized to the pulmonary region. Through local multiplication and entry into the bloodstream, disseminated MAC infections may complicate the pulmonary disease by spreading to other organs and tissues. This disease is common in advanced HIV patients, patients with a history of immunosuppressive therapy, and other severely immunocompromised patients [24,79] (p. 13).

Treatment of MAC infection for patients with AIDS involves a combination of antimicrobial and antiretroviral therapy (ART) and may take more than 12 months. A dual therapy with a macrolide, azithromycin (500–600 mg daily), or clarithromycin (500 mg twice daily), combined with ethambutol (15 mg/kg daily) is initially used. In patients with failing ART, and when the mycobacterial counts in blood culture are high, a third agent (e.g., rifabutin) is added [80,81].

#### 2.4.3. MAC Lymphadenitis

Nontuberculous mycobacterial (NTM) and MAC-specific lymphadenitis is transmitted through environmental sources and has a high risk of incidence among immunodeficient children [82,83]. The treatment of NTM or MAC specific lymphadenitis includes surgical excision and/or antimicrobial therapy depending on whether or not patients are good candidates for surgical excision. The increased risk of facial nerve damage can be a significant determining factor in whether to use antimicrobial therapy instead of surgical excision [84]. However, for patients with NTM lymphadenitis and no evidence of disseminated disease, surgical excision is the initial intervention and provides the optimal specimen for diagnostic testing. Antimicrobial therapy, with or without subsequent tissue excision, is the suggested method of therapy. An observational method of therapy is also sometimes utilized and involves monitoring patients for spontaneous disease resolution without the use of therapies. This prolonged course of observational treatment can be difficult to tolerate for some families [85]. The suggested regimen for antimicrobial therapy includes a macrolide in combination with ethambutol and/or rifampin. Azithromycin is the preferable macrolide for children due to it being more palatable and dosed once daily [5]. The duration for antimicrobial therapy of NTM or MAC specific lymphadenitis may take up to six months depending on the resolution of symptoms [86].

## 3. Mycobacterium abscessus

### 3.1. General Characteristics

*Mycobacterium abscessus* are NTM under Runyon group IV, classified as rapid-growing mycobacteria (RGM). Similar to most NTM species, these organisms are ubiquitous in the environment and have been found in soil, natural and drinking water sources, sewage water, as well as decaying vegetation [87]. They are typically non-pathogenic but are emerging as opportunistic pathogens that can cause a wide range of clinical diseases such as pulmonary lung disease, skin and soft tissue infections, including in the cartilage, tendons, and the layers of fat and muscle under the skin [5,87]. In Japan and America, approximately 5% of pulmonary infections are due to RGM and about 65–80% of the RGM infections are due to *M. abscessus* [88]. Pulmonary disease caused by *M. abscessus* is common among cystic fibrosis (CF) patients, while the disseminated infection is common among immunocompromised patients. Soft tissue infections are often healthcare-associated infections due to open wounds being contaminated with non-sterile medical or surgical equipment or materials. Infection can also occur after a wound is contaminated with soil [89]. Thus, pulmonary disease and disseminated infection are associated with patients with preexisting conditions, while soft tissue or skin infections are associated with post-surgical or healthcare-associated infections [89].

*M. abscessus* is one of the most resistant organisms to chemotherapeutic agents and its main threat as a human pathogen is due to its high multi-drug resistance. This bacterium is also associated with biofilm formation, resistance to disinfectants, high temperatures, and acidic environments. Previously, *M. abscessus* was thought to be acquired exclusively from the environment but the use of whole-genome sequencing (WGS) analysis from worldwide clinical isolates of *M. abscessus* suggested there was possibly human-to-human transmission of *M. abscessus* [90].

*M. abscessus* was first isolated in 1953. Since then, *M. abscessus* has displayed a high degree of genotypic heterogeneity among subspecies; identified through genome sequencing of molecular targets such as hsp65 and rpoB [91,92,93]. The *M. abscessus group* is divided into three subspecies: *M. abscessus* subsp. *abscessus; M. abscessus* subsp. *bolletii* and *M. abscessus* subsp. *massiliense*, which for the rest of the paper will be referred to as *M. abscessus, M. bolletii, and M. massiliense*, respectively. Both *M. abscessus* and *M. bolletii* have functional isolates of the erythromycin ribosomal methylase (*erm*)41 gene that gives them inducible macrolide resistance compared to *M. massiliense* which does not have a functional *erm* (41) gene. This inducible resistance, therefore, explains the better response to treatments of *M. massiliense* infections compared to *M. abscessus and M. bolletii* infections [94]. However, there are recent reports of the full length *erm* gene in *M. massiliense* isolates that confuses this previous identification of *M. massiliense* due to not having a functional *erm* (41) gene [95]. The nonfunctional *erm* (*41*) gene has also been found in some isolates of *M. abscessus* subsp. *abscessus* due to a C instead of T at the nucleotide position 28 in the *erm* (41) gene [96,97]. In addition to genotypic heterogeneity, there is also phenotypic heterogeneity among the *M. abscessus* organisms that contribute to its virulence [98]. Two distinct morphotypes exist depending on the presence of glycopeptidolipids (GPL) in the cell wall. Smooth variants have GPL present, while GPL is absent in the cell walls of rough variants [98]. Other NTM species also display this morphological heterogeneity.

### 3.2. Vulnerable Populations to Mycobacterium abscessus Infections

*M. abscessus* is categorized as a rapidly growing mycobacterium (RGM) within the NTM species, demonstrating disseminated infections in immunocompromised, trauma, and postsurgical patients [99]. Similar to other NTM, they are widely dispersed throughout the environment, with *M. abscessus* isolates found in freshwater rivers and lakes, seawater, and animal drinking troughs, as well as peat-rich potting soils. The survival and growth of pathogenic *M. abscessus* have been recovered in large quantities within our water distribution systems such as drinking water, household plumbing, and even hospital wastewater [100,101]. *M. abscessus* has a hydrophobic outer surface, leading to biofilm formation and resistance to disinfectants and antibiotics [101]. Bacteria can enter the body through damaged skin, such as injured wounds, ulcerations, via contaminated soil and water [99,101]. The majority of infections in healthy patients usually follow penetrating trauma and subsequent inoculation of *M. abscessus* of the host tissue barrier [89]. Numerous soft tissue infections have been derived from naturally occurring puncture wounds, fluid-contaminated injections, and postoperative surgical care [89]. Healthcare-associated infections (HAI) have also been reported following micrographic and laser surgery, suggesting that mild trauma during these operations predisposes even immunocompetent patients to disease [89]. Patients with impaired lung tissue or airways from emphysema, cystic fibrosis, or a previous tuberculosis infection, are also vulnerable to *M. abscessus* infections [102].

Patients suffering from cystic fibrosis (CF) have an increased chance of colonization and infection from *M. abscessus* [103]. Prospective studies have demonstrated CF as one of the leading risk factors for NTM infections, with *M. abscessus* by far being the most prevalent [104,105]. The isolation of *M. abscessus* has been correlated with declining lung function among CF patients [106]. For example, a pediatric patient suffering from CF with a pulmonary *M. abscessus* infection deteriorated from only suffering from minimal bronchiectasis to requiring a lung transplantation procedure within a period of 29 months [103]. Another CF patient died from a devastating *M. abscessus infection* in connection with a pulmonary transplant, likely generated from her originally infected lungs [107]. These observations are concerning and display that *M. abscessus* is a pathogen that may induce serious infection risks for CF patients. The dangers of *M. abscessus* complex infections in hospitals relating to lung transplantations and cystic fibrosis are concerning to medical professionals, and these dangers are compounded by the risk of nosocomial infections that have already occurred in the past [102].

People with immunosuppressed diseases such as HIV or people who have an organ implant are also vulnerable. As mentioned earlier, in HIV patients, disseminated infection does not usually develop unless the CD4+ T-lymphocyte count is below 50/mm^3^; however, disseminated *M. abscessus* infection is rarer [108]. Patients with gastroesophageal reflux disease (GERD) can also develop an NTM-related lung infection if they accidentally inhale water contaminated with NTM [109]. Bronchiectasis, aspiration, and chronic vomiting are also associated with pulmonary *M. abscessus* infections [110,111]. In addition, some patients with certain polymorphisms such as the natural-resistance-associated macrophage protein 1 are increasingly susceptible to pulmonary *M. abscessus* infection despite no underlying lung disease [66]. Overall, environmental *M. abscessus* demonstrates the potential to be a pathogen in hosts with a tissue barrier function that is compromised, hosts with airway abnormalities, and in immunodeficient patients.

### 3.3. Pathogenesis of Mycobacterium abscessus

#### 3.3.1. The Relation of Morphotypes and Pathogenesis

*M. abscessus* is the most virulent RGM and the cause of most pulmonary infections due to RGM. NTM infection involves a complex interaction between the host, pathogen, and environment. Typically, *M. abscessus* pulmonary infections demand the most challenging treatments out of all NTM infections. It shares many pathophysiological symptoms with *M. avium* and *M. tuberculosis*, such as the formation of granulomas and enduring infection; however, the specific pathways in the establishment of infection and the exact routes of transmission are not well understood. There is evidence that colonization of the lung airways is facilitated by bacterial biofilm formation, with glycopeptidolipids playing an important role. In NTM, including *M. abscessus*, glycopeptidolipids (GPLs) are found in the outer layer of the cell envelope. The expression of GPL is correlated with the smooth colony phenotype, while a lack of GPL results in a rough colony [112]. Smooth colonies have a morphology that is round, uniform, and glossy, while the rough colonies are irregular, corded, and dry. *M. abscessus* complex can display both colony morphotypes.

Smooth and rough colonies not only have different physical appearances but have different pathophysiological traits. The ability to switch between smooth and rough morphologies allows *M. abscessus* to transition between a colonizing phenotype and a more virulent and invasive form [113]. Rough variants were found to persist in the lungs of Severe Combined Immunodeficiency (SCID) mice, replicates in macrophages, and formed corded invasive microcolonies in fibroblast monolayer, while the smooth variant did not display the same characteristics [114]. GPL in the cell wall of smooth variants was found to give the smooth variant the ability of biofilm formation and sliding mobility [115,116]. It was observed that smooth variants were able to establish biofilm in the lung airways of patients with abnormal lung airways and diminished mucociliary clearance. A mutant that lacked GPL and exhibited the rough phenotype was shown to spontaneously arise from the smooth phenotype and gain virulence characteristics [115]. Studies indicate that clinical isolates from individuals with an infection exhibit the rough phenotype [117]. The ability for *M. abscessus* to change from smooth to rough variants was associated with the increased severity of clinical symptoms [118]. It has been shown that GPL masks the underlying phosphatidyl-myo-inositol mannosides (PIMs) interfering with pathogen recognition by the host’s innate immune system [112]. All these observations indicate that *M. abscessus* initially gains entry to the lungs as a GPL-expressing smooth variant, and then the spontaneous loss of GPL expression results in a virulent phenotype causing inflammation and invasive lung disease.

Macrophages can take up both smooth and rough variants of *M. abscessus*. GPL-rich smooth bacilli are taken up by macrophages individually and maintained in loner phagosomes [119]. In contrast, GPL-deficient rough bacilli are phagocytized by the macrophage within small clusters resulting in the creation of social phagosomes, where a large number of bacteria are in one phagosome [119]. The smooth variants in loner phagosomes are kept close between the phagosomal membrane and the bacterial surface, establishing the electron transparent zone (ETZ) [120]. The ETZ prevents the maturation of phagosomes, apoptosis-mediated pathways, and the activation of autophagy. The smooth variants can survive for an extended period within a macrophage and ultimately recruit phagocytes in close proximity to start forming a granuloma. On the other hand, the rough variant in the filled social phagosome melds with the lysosome, causing the acidification of the phagosome, autophagy activation, and apoptosis [119]. Due to the strong induction of macrophage apoptosis, bacilli are released into the extracellular environment, freely replicating and ultimately forming massive cord-like structures. One study showed that extracellular cording allowed the mycobacteria to prevent phagocytosis, resulting in uncontrolled growth, extensive tissue destruction, and strong inflammation. The massive production of cords, which is absent in the smooth variants, allows the bacteria to establish the infection and is a determinant of virulence [121].

#### 3.3.2. Host Immune Response

Macrophages infected with smooth *M. abscessus* produce little pro-inflammatory cytokines and control infection, as opposed to macrophages infected with the rough variant, produce copious amounts of pro-inflammatory cytokines, with the bacteria surviving and growing both intracellularly and extracellularly [115,117,122,123]. Rough variants lacking GPL stimulate the innate immune response in respiratory epithelial cells via toll-like receptor 2 (TLR2), while the GPL-expressing smooth *M. abscessus* do not. The TLRs mediate the innate immune response, recognizing conserved motifs expressed by the microorganisms, called pathogen-associated molecular patterns (PAMPs) [124]. TLRs are present on macrophages, dendritic cells (DCs), and respiratory epithelial cells in the lining of the lungs. The expression of phosphatidyl-myo-inositol mannosides’ (PIMS) surface molecules on *M. abscessus* have been shown to stimulate the macrophage immune response via TLR2 signaling [112]. Stimulation of TLR2 also results in the gene expression of downstream effector molecule human beta-defensin 2 (HBD2) and the release of IL-8 [125]. *M. abscessus* rough variants also stimulate the release of TNF-α via interaction with TLR2. The release of IL-8 is also involved in the recruitment of neutrophils to the site of infection in the lung. Neutrophil extracellular trap (NET) formation, reactive oxygen species (ROS) generation, and phagocytosis are some common neutrophil mechanisms activated by both smooth and rough variants [126]. In murine macrophages, it was shown that transient interaction between TLR2, pattern recognition receptors, and dectin-1 on cell surfaces resulted in the induction of cytokines, TNF-α, IL-6, and p40 subunit of IL-12, and enhanced phagocytosis [124].

Several studies report that mitogen-activated protein kinases (MAPKs), specifically ERK1/2 and p38 are key modulators for granuloma formation during an NTM infection [127]. This is consistent with one study demonstrating MAPKs’ activation in *M. abscessus* MAB2560-treated DCs. Another finding from the same study suggests that *M. abscessus* MAB2560 enhanced DC maturation via TLR4 and activated MAPKs’ production of IL-12 [128]. The secretion of IL-12 will then activate and polarize naive CD4+ T cells towards Th1 cells to produce IFN-γ. Macrophages and NK cells can both release IL-12 and IFN-γ to guide T cells to the Th1 type phenotype [129]. Both IFN-γ and TNF-α are essential in the host defense against *M. abscessus*, as genetic deficiencies in TNF-α receptor, TFN-γ, or CD4+ cause increased susceptibility.

### 3.4. Treatment of Mycobacterium abscessus Infections

Complex *M. abscessus* infections are extremely difficult to treat due to their high antimicrobial drug and disinfectant resistance [130]. *M. abscessus* complex infections mainly result in skin and soft tissue infections (SSTIs) and pulmonary disease, but can also cause ocular and central nervous system (CNS) infections [5,130].

*M. abscessus* complex-associated pulmonary disease is one of the most common and well-known NTM infections. There is no standard treatment, but current guidelines recommend a macrolide-containing multidrug treatment regimen with intravenous antimicrobial agents, although this therapy is known to have considerable side effects [5]. This multidrug regimen should include at least three active drugs, guided by in vitro macrolide susceptibility testing in the initial treatment phase [67]. As previously mentioned, it has been found that both *M. abscessus* and *M. bolletii* have functional isolates of the erythromycin ribosomal methylase (*erm*)41 that gives these subspecies inducible macrolide resistance compared to *M. massiliense*, which has a nonfunctional *erm* (*41*) gene. Although this is mainly the case, the nonfunctional *erm*(*41*) gene has occurred in some *M. abscessus* subsp. *abscessus* due to a C instead of T mutation at the nt position 28 in the *erm*(*41*) gene [96,97]; recent reports of the full length *erm* gene in *M. massiliense* isolates further confuse this previous identification [95]. Macrolides are very active in vitro against strains without a functional *erm* (*41*) gene; therefore, it is important to conduct in vitro susceptibility testing to detect a functional or nonfunctional *erm*(*41*) gene. All three subspecies can also develop constitutive macrolide resistance due to 23S RNA (*rrl*) gene mutations as well [97]. Similar to the treatment of MAC infections, the CLSI strongly advises for identification to the subspecies level for *M. abscessus* complex and recommends antimicrobial susceptibility testing (AST) on all clinically significant isolates. Susceptibility panels for *M. abscessus* need to include at least amikacin, cefoxitin, imipenem, clarithromycin, linezolid, doxycycline, tigecycline, ciprofloxacin, and moxifloxacin [67]. These guidelines for AST are important for the proper selection of antimicrobial treatment regimens for *M. abscesses*, as well as MAC and other NTM pathogens [72].

Based on the guidelines from the American Thoracic Society/Infectious Diseases Society of America in 2007, treatment with current existing antimicrobial agents is limited [5]. Given the variable and limited in vitro susceptibility of these organisms, the potential for drug resistance, and the rapid progression of *M. abscessus* pulmonary disease, treatment regimens should be developed in collaboration with experts [67]. Additionally, there is no optimal duration of therapy and it could be shorter or longer based on expert consultation. Although many medication regimens show efficacy in non-pulmonary disease, there are no antibiotic regimens based on in vitro susceptibilities that have shown to result in long-term sputum conversion for *M. abscessus* lung disease. Suppressive therapy, with periodic parenteral antibiotic or orally administered macrolide therapy, can be administered to control the progression and symptoms of the disease. Surgical resection of the infected lung following chemotherapy to lessen the microbial burden is a likely curative therapy for *M. abscessus* lung disease [3]. Compared to antimicrobial treatment alone, surgical resection of the localized infection in combination with antimicrobial therapy has proven to have better outcomes. Indications for *M. abscessus* lung disease, similar to all NTM lung disease, include a poor response to medical treatment for at least 6 months, if patients have sufficient cardiopulmonary function to withstand and tolerate the surgery. The outcomes have been shown to be successful, but with relatively high complication rates [71].

The *M. abscessus* complex can also cause SSTIs, ranging from deep tissue to partial skin infections. The two main mechanisms for developing *M. abscessus*-associated SSTIs are from (1) direct contact with a traumatic injury or surgical wound, with contaminated water, materials, or environmental exposure and (2) through a secondary infection of the skin and soft tissue associated with disseminated disease [99]. It has been reported that undergoing cosmetic procedures like mesotherapy, tattooing, and acupuncture can cause *M. abscessus*-associated SSTIs [99]. Exposure to *M. abscessus* from spas or hot springs can also result in SSTIs [99,131]. It is important to note that SSTIs among hospitalized post-surgical patients are predominately caused by the *M. massiliense subspecies* [132,133]. For serious *M. abscessus* complex skin, soft tissue, or bone infections, the initial recommendation for treatment includes a combination of antimicrobial drugs with a macrolide (clarithromycin with 1,000 mg daily or azithromycin with 250 mg to 500 mg daily) and intravenous agents for between two weeks and several months after oral macrolide-based therapy [3]. The initial intravenous drug treatment is amikacin for (25 mg/kg 3×/week) and cefoxitin (up to 12 g/d in divided doses) or amikacin (25 mg/kg 3×/week) and imipenem (500 mg 2–4×/week). In vitro studies showed tigecycline had low MICs and should also be considered in treatment regimens. Surgery is generally performed when the disease progresses extensively, where drug intervention is difficult and an abscess forms. [3].

Although it is rare, *M. abscessus* can also cause CNS infections, with cerebral abscesses and meningitis being the most common. Among HIV-positive patients, MAC accounts for more CNS infections, while *M. abscessus* is responsible for more CNS infections among HIV-negative patients. HIV-negative patients that acquired *M. abscessus*-associated CNS infections are often patients with recent neurosurgical procedures or intracranial catheters. Treatment includes at least one year of clarithromycin-based combination therapy and surgical intervention if needed [132]. The treatment outcomes are based on the individual’s health condition.

Ocular infections caused by NTM have also increased over the past decade. *M. abscessus* complex ocular infections were initially controlled by discontinuation of topical corticosteroids. Currently, optimal treatment methods depend on the location of the ocular infection [134]. Systemic antimicrobial drugs can be used to treat most *M. abscessus*-associated ocular infections, while topical therapy with topical amikacin and clarithromycin is used to treat only certain *M. abscessus* complex ocular infections [134]. Surgical debridement of infected tissue should also be utilized for some patients [134]. Treatment outcomes depend on the location of the infection and how early it is recognized.

Co-infection of MAC and *M. abscessus* has an uncertain clinical significance that is further complicated by difficultly treating *M. abscessus* infections. There is not yet an effective or convenient dual therapy for co-infection [5]. MAC therapy is not adequate for *M. abscessus* treatment, and the need for parenteral antibiotics that are slow and unpredictable for *M. abscessus* infections are daunting for clinicians to expose prolonged toxic medication to patients [135,136]. Currently, there have not been enough data to make conclusions about the optimal treatment for patients with co-infection with multiple mycobacterial species, such as MAC and *M. abscessus* complex (MABC).

## 4. Diagnosis of NTM Lung Disease

Presumptive diagnoses based on clinical and radiographic features are not sufficient for the initiation of therapy; there must be an isolation of NTM species through microbiologic cultures. Additionally, since NTM species are widely found in nature and tap water contamination is highly possible; NTM disease diagnosis requires three positive sputum cultures collected on three separate days. Radiographic tests, when compared to TB disease, predominately show NTM disease to appear as (1) thin-walled cavities with less surrounding parenchymal opacity, (2) less bronchogenic but more contiguous spread of infection, and (3) more pronounced involvement of pleura over the affected areas of the lungs [5,137,138]. *M. abscessus* lung disease in patients has a very similar pattern in radiographic appearance to the nodular bronchiectatic form of MAC lung disease; additionally, 15% of patients with *M. abscessus* lung disease will also have MAC species isolated in the same sputum. However, TB disease cannot be ruled out based on radiographic appearance alone; therefore, clinical and microbiological criteria must also be met for the diagnosis and initiation of treatment [5]. According to the American Thoracic Society, in their official ATS/IDSA statements, the diagnostic criteria for NTM lung disease, when applied to symptomatic patients, include a minimum of the following: (1) chest radiograph or, in the absence of cavitation, high-resolution computed tomography (HRCT) chest scan; (2) three or more sputum specimens for acid-fast bacilli (AFB) analysis; and (3) exclusion of other diseases, such as tuberculosis (TB). Clinical, radiographic, and microbiologic criteria are all equally important and a diagnosis of NTM lung disease requires all three [5]. See Table 3 below, which has been adapted from the ATS/IDSA official statement published in 2007.

## 5. Prevention

Exposure to NTM can come from household and hospital water systems, hemodialysis centers, and dental clinics [139]. Animals may also be a reservoir and sharing water systems should be avoided [140]. Improper disinfection of medical equipment in the hospital, such as bronchoscopes and endoscopes, can also lead to NTM colonization and infection. The prevention of NTM infections is challenging due to mycobacteria’s high lipid content and triple-layered wall, making them more resistant to disinfectant, elevated temperatures, and ultraviolet light in comparison to other bacteria that may colonize water systems. Additionally, NTM commonly exists in the biofilm that coat water system pipes and fixtures that make them more resistant to disinfectant. Precautions should be taken by requiring high-level disinfectant on any instruments that will be in contact with mucous membranes [1]. The installation of filters and the periodic flushing of water systems may also reduce the passage of NTM [139]. There is also evidence that bathroom shower heads can aerosolize NTM [141]. Measures can be taken by increasing hot water temperature, reducing the mist in the showers by increasing the diameter of water streams, and installing filters that prevent the passage of NTM. The avoidance of hot tubs, especially in public, may also prevent the infection of skin wounds [142].

HIV-positive adults are at an increased risk of disseminated MAC infection and should receive primary prophylaxis for certain indications [143]. If HIV-positive patients are beginning ART, primary prophylaxis is not recommended. However, HIV-positive patients who are not receiving ART should be given primary prophylaxis with macrolides. Once patients are on a fully suppressive antiretroviral therapy (ART) regimen, they can discontinue the macrolide prophylaxis with minimal risk of developing a disseminated MAC infection [144,145]. Indications to restart primary prophylaxis are if CD4 cell counts are below 50 cells/mm^3^ and if the patient is not on fully suppressive ART [146]. After treatment of an existing disseminated MAC infection, HIV patients can be put on chronic maintenance therapy or secondary prophylaxis to prevent reoccurrences. Secondary prophylaxis should only be indicated for patients who cannot be on a fully suppressive ART regimen and who consistently have levels of CD4 cells below 100 cells/mm^3^ [146]. Long-term macrolide therapy can also be used for patients with lung diseases such as bronchiectasis or cystic fibrosis to reduce exacerbations including NTM infections. The long-term use of macrolides should be approached with caution, as there are limited data supporting an increase in macrolide resistance. However, there are limitations to the existing data and longer term studies should be done on macrolide resistance for long-term use [147]. Rifabutin, clarithromycin, and azithromycin are common prophylactic agents used for MAC in people with HIV/AIDS [148,149]. Prophylaxis has been less studied systemically for other NTM species [150]. Patients undergoing lung transplants should consider pretransplant or posttransplant chemoprophylaxis, depending on their condition and medical history. Patients with cystic fibrosis, who are known to be colonized by RGM, should be considered for post-transplant chemoprophylaxis, while patients with past NTM infections should be considered for multidrug MAC therapy before lung transplants [151].

BCG vaccination may also be a prevention strategy, with one study showing that children with a history of bacillus Calmette–Guerin (BCG) vaccination appear to have a reduced risk of MAC lymphadenitis [152]. The prevention of *M. abscessus* infection specifically should include improved protocols for the hygiene of medical professionals and patients such as handwashing, the wearing of personal protective equipment (PPE), disinfecting surfaces and equipment, and the sterilization of any tools that are used for invasive procedures. Due to the high resistance of *M. abscessus* complex to disinfectants, bleach in combination with another disinfectant should be used to disinfect surfaces [153]. Preventing infections for patients with lung transplants, cystic fibrosis, or a compromised immune system is much more complicated. To prevent nosocomial transmission of *M. abscessus* complex in these at-risk groups, negative pressure ventilation systems for each inpatient room are recommended, as well as optimizing the air exchange rates of the rooms and communal areas [153]. The implementation of the mentioned measures can reduce the risk of exposure to NTM, and recommendations should be given to people, especially vulnerable patients with recognized risk factors such as immunocompromised patients or patients who have other underlying health conditions.

## 6. Summary

Nontuberculous mycobacterium (NTM), including over 172 distinct species, are a group of ubiquitous environmental bacteria found in soil and water sources. Most NTM species are non-pathogenic; however, a few species cause opportunistic human infections. Slow-growing mycobacteria, *Mycobacterium avium*, and rapid-growing mycobacteria, *Mycobacterium abscessus*, are important emerging human pathogens causing disease in vulnerable populations such as severely immunocompromised patients, patients with chronic lung diseases, and others with underlying health conditions. Their incidence is increasing worldwide in industrialized countries and they cause substantial yet underacknowledged burden of disease. Within the context of *Mycobacterium avium* complex (MAC), *M. avium* causes progressive pulmonary disease, disseminated infection, and lymphadenitis. *M. abscessus* is a highly pathogenic and drug-resistant fast-growing mycobacterium and is responsible for most pulmonary infections caused by RGM. The pathogenesis of M. *avium* involves its ability to colonize and invade intestinal or respiratory mucosa to infect phagocytic cells. The pathogenesis of *M. abscessus* is related to its ability to form biofilms and transform from a colonizing GPL-expressing smooth phenotype that evades innate immune responses to a virulent GPL-deficient rough phenotype, capable of causing extensive tissue damage. Both infections often require a combination of therapies that can include antimicrobial therapy, particularly macrolides, ART therapy in HIV patients, and sometimes surgical excision. The prevention of NTM infection may include the installation of filters and periodic flushing of water systems, and improved protocols for hygiene and the disinfection of medical devices. These recommendations should be shared with people, especially vulnerable patients with recognized risk factors such as immunocompromised patients or patients who have underlying health conditions.

## Figures and Tables

**Table 1:** Human pathogenic mycobacteria.

Human Pathogenic Mycobacteria				
Group	Mycobacterium Tuberculosis Complex	Non-Tuberculous Mycobacteria		Leprotic Mycobacteria
		Rapidly Growing	Slow Growing	
**Species**	*M. tuberculosis* *M. bovis* *M. africanum* *M. canettii* *M. caprae* *M. pinnipedii* *M. orygis* *M. microti* *M. mungi* *M. suricattae*	*M. abscessus group* *-M. abscessus* *-M. bolletii* *-M. massiliense* *M. fortuitum group* *-M. fortuitum* *-M. peregrinum* *-M. porcinum* *M. smegmatis* *M. vaccae* *M. mucogenicum*	*M. avium complex* *-M. avium* *-M. chimaera* *-M. intracellulare* *M. haemophilum* *M. gordonae* *M. kansasii* *M. simiae* *M. marinum* *M. malmoense* *M. xenopi* *M. ulcerans*	*M. leprae* *M. lepromatosis*

**Table 2:** Recommended treatment regimen.

	Type of Disease	Recommend Regimen
*Mycobacterium avium* Disease	Pulmonary MAC Infections	macrolides, followed by close follow-up monitoring for up to a year
	Mild or moderate bronchiectatic disease	azithromycin (500 mg three times per week), rifampin (600 mg three times per week), and ethambutol (25 mg/kg three times per week)
	Cavitary or severe nodular bronchiectatic disease	a daily regimen of azithromycin (250 to 500 mg daily), rifampin (600 mg daily), and ethambutol (15 mg/kg daily) and a fourth agent consisting of parenteral streptomycin or amikacin (10 to 15 mg/kg three times per week) is used for the first 8 to 12 weeks of therapy
	Macrolide resistant infections	daily ethambutol, rifampin, and clofazimine, in addition to two to three months of parenteral amikacin administered three times a week
	Disseminated MAC infections with AIDS	combination of antimicrobial and antiretroviral therapy (ART) and may take more than 12 months; dual therapy with a macrolide, azithromycin (500–600 mg daily), or clarithromycin (500 mg twice daily), combined with ethambutol (15 mg/kg daily) is initially used
	Disseminated MAC infections with AIDS failing ART	dual therapy with a macrolide, azithromycin (500–600 mg daily), or clarithromycin (500 mg twice daily), combined with ethambutol (15 mg/kg daily) is initially used, plus a third agent (e.g., rifabutin) is added
	MAC Lymphadenitis	surgical excision and/or antimicrobial therapy; antimicrobial therapy includes a macrolide in combination with ethambutol and/or rifampin; Azithromycin is the preferable macrolide for children; the duration for antimicrobial therapy of NTM or MAC specific lymphadenitis may take up to six months
Mycobacterium abscessus Diseases	M. abscessus complex-associated pulmonary disease	combination of macrolide-based therapy with intravenous antimicrobial agents; surgical resection of the localized infection in combination with antimicrobial therapy; continue until sputum samples are negative for M. abscessus complex for 12 months
	M. abscessus complex-associated skin and soft tissue infections (SSTIs)	macrolide in combination with amikacin plus cefoxitin/imipenem plus surgical debridement; minimum of 4 months, including a minimum of 2 weeks combined with intravenous agents
	M. abscessus-associated central nervous system infections: cerebral abscesses and meningitis	treatment includes at least one year of clarithromycin-based combination therapy (preferably including at least amikacin in the first weeks) for 12 months and surgical intervention if needed
	M. abscessus complex ocular infections	systemic antimicrobial drugs can be used to treat most M. abscessus-associated ocular infections, while topical therapy with topical amikacin and clarithromycin are used to treat certain M. abscessus complex ocular infections for 6weeks to 6 months
	Serious M. abscessus complex infections	initial treatment should include a combination of antimicrobial drugs with a macrolide (clarithromycin with 1,000 mg daily or azithromycin with 250 mg to 500 mg daily) and intravenous agents for two weeks to several months subsequently after oral macrolide-based therapy. The initial intravenous drug treatment is amikacin for (25 mg/kg 3×/week) and cefoxitin (up to 12 g/d in divided doses) or amikacin (25 mg/kg 3×/week) and imipenem (500 mg 2–4×/week)

**Table 3:** Radiological and microbiologic criteria for nontuberculous mycobacteria (NTM) lung disease diagnosis.

Test Type	Criteria Required
Clinical and/or Radiological	1. Pulmonary symptoms, nodular or cavitary opacities on chest radiograph, or an HRCT scan that shows multifocal bronchiectasis with multiple small nodules. AND 2. Appropriate exclusion of other diagnoses. (* both are required)
Microbiologic	1. Positive culture results from at least two separate expectorated sputum samples. (If the results from the initial sputum samples are non-diagnostic, consider repeat sputum AFB smears and cultures.) OR 2. Positive culture results from at least one bronchial wash or lavage. OR 3. Transbronchial or another lung biopsy with mycobacterial histopathologic features (granulomatous inflammation or AFB) and positive culture for NTM or biopsy showing mycobacterial histopathologic features (granulomatous inflammation or AFB) and one or more sputum or bronchial washings that are culture positive for NTM. 4. Expert consultation should be obtained when NTM are recovered that are either infrequently encountered or that usually represent environmental contamination. 5. Patients who are suspected of having NTM lung disease but who do not meet the diagnostic criteria should be followed until the diagnosis is firmly established or excluded. 6. Making the diagnosis of NTM lung disease does not, per se, necessitate the institution of therapy, which is a decision based on potential risks and benefits of therapy for individual patients.

* Table 3 is adapted from The Official Statement of the American Thoracic Society (ATS) and the Infectious Diseases Society of America (IDSA) for Diagnosis, Treatment, and Prevention of Nontuberculous Mycobacterial Diseases, 2007.
